# Regulation of surfactant protein D in the rodent prostate

**DOI:** 10.1186/1477-7827-5-42

**Published:** 2007-11-07

**Authors:** Rebecca E Oberley, Kelli L Goss, Amado A Quintar, Cristina A Maldonado, Jeanne M Snyder

**Affiliations:** 1 Department of Medicine, National Jewish Medical and Research Center, Denver, USA, CO 80206; 2 Department of Anatomy and Cell Biology, University of Iowa College of Medicine, Iowa City, Iowa, USA, 52242; 3 Center for Electron Microscopy, School of Medical Science, National University of Cordoba, Cordoba, Argentina

## Abstract

**Background:**

Surfactant protein D (SP-D) is an innate immune protein that is present in mucosal lined surfaces throughout the human body, including the male reproductive tract. In the present study, we characterized the regulation of SP-D expression in the mouse and rat prostate.

**Methods:**

Real time reverse transcriptase polymerase chain reaction (RT-PCR) and immunostaining were used to characterize SP-D mRNA and protein in the mouse male reproductive tract. In order to evaluate the effects of testosterone on SP-D gene expression, we measured SP-D mRNA levels via real time RT-PCR in prostates from sham-castrated mice and castrated mice. In addition, we used a rat prostatitis model in which Escherichia coli was injected into the prostate in vivo to determine if infection influences SP-D protein levels in the prostate.

**Results:**

We found that SP-D mRNA and protein are present throughout the mouse male reproductive tract, including in the prostate. We determined that castration increases prostate SP-D mRNA levels (~7 fold) when compared to levels in sham-castrated animals. Finally, we demonstrated that infection in the prostate results in a significant increase in SP-D content 24 and 48 hours post-infection.

**Conclusion:**

Our results suggest that infection and androgens regulate SP-D in the prostate.

## Background

Although nonbacterial prostatitis is more common, prostatitis can be caused by bacterial infection that over time may lead to inflammation of the prostate [1]. Moreover, patients with chronic prostatitis have important alterations in several measures of semen quality and it has been proposed that prostatitis may contribute to male infertility [2]. In spite of this, relatively little is known about innate immune defense mechanisms within the prostate gland. *Escherichia coli *is a pathogen frequently associated with both acute and chronic bacterial prostatitis in humans [3,4]. Although bacteria have not been definitively shown to cause benign prostate hyperplasia (BPH), a relatively common prostate disease, *E. coli *has been implicated in its pathogenesis [5]. It has been speculated that chronic low-grade colonization with *E. coli *can cause prostate pathology via the release of endotoxin over a long period of time and this, in concert with dihydrotestosterone (DHT), can lead to hyperplasia of the prostate [5]. For decades, it has been known that seminal plasma and prostatic fluid have antimicrobial properties [6,7]. Recently, several agents responsible for this activity have been identified and characterized in the male reproductive tract [8].

Surfactant protein D (SP-D) is a member of the collectin family of proteins which play an important role in innate immune responses [9]. SP-D can act as an opsonin to increase the phagocytosis of a variety of pathogens [10-12]. SP-D also promotes the phagocytosis of pathogens via direct interactions with macrophages and neutrophils [10,13]. In contrast, SP-D can inhibit the uptake of some pathogens, for example *Candida albicans *and *Mycobacterium tuberculosis *[14,15]. Recently, SP-D has been shown to interact with the adaptive immune system by enhancing bacterial antigen presentation to dendritic cells [16]. Finally, SP-D can have direct anti-microbial effects by disrupting bacterial membranes [17].

SP-D was originally described in the lung, but recent studies have shown that SP-D is expressed throughout the body, usually at mucosal surfaces [18,19][20,21][22,23]. For example, SP-D has been detected in the digestive tract as well as in the reproductive tract of the mouse, rat and human species [13-18]. We have recently reported that SP-D is present in the human prostate and that SP-D protects prostate epithelial cells from infection by *Chlamydia in vitro *[23].

SP-D is expressed constitutively in the lung and pulmonary SP-D levels can vary with infection or disease [24]. Lung SP-D levels are decreased in cystic fibrosis and acute respiratory distress syndrome [24]. In contrast, lung SP-D gene expression has been shown to be increased 24–72 hours after intratracheal instillation of lipopolysaccharides (LPS) [25]. Challenge with *Pseudomonas aeruginosa*, influenza virus, or the overexpression of cytokines such as interleukin-4 can also lead to elevated SP-D levels in the lung [26-28].

In the present study, we found that SP-D mRNA and protein are expressed throughout the mouse male reproductive tract. We then showed that castration increases SP-D mRNA levels in the prostate. We also demonstrate that infection of the rat prostate with *E. coli *leads to an increase in SP-D protein levels in the prostate gland 24 to 48 hours post infection. These data are suggestive that SP-D produced in the prostate is regulated by androgens and influenced by infection.

## Methods

### Animal husbandry

Swiss Black mice were obtained from Taconic Laboratories (Hudson, NY) and bred at the Animal Research Facility at the University of Iowa following an approved protocol. Sham-castrated or castrated Swiss Black mice were also obtained from Taconic Laboratories. All mice were housed under pathogen-free conditions and allowed food and water *ad libitum*.

Adult, twelve-week old male Wistar rats were housed at an animal research facility in the Universidad Nacional de Cordoba, Cordoba, Argentina. The rats were housed under controlled conditions of 14 hours of light followed by 10 hours of darkness and allowed food and water *ad libitum*. All rat experimental protocols were performed in accordance with NIH guidelines for animal care.

### Castration and sham-castration experiments

Castrated and sham-castrated Swiss Black mice were obtained from Taconic Laboratories. The animals were housed for 12 days after surgery to ensure that testosterone was completely cleared. All animals, i.e., sham-castrated and castrated, were then sacrificed and the prostate (ventral and dorsal) and lungs removed. In some cases the epididymis and bladder were removed as well. This experiment was repeated three times with 3 animals per condition (a total of 9 animals per group).

### Bacterial prostatitis model

We wished to determine if SP-D protein expression in the prostate is altered with infection. A prostatitis model, achieved via direct infection with *E. coli*, was used to address this question. We chose to perform these studies in the rat because of the greater size of the rat prostate, which enables easier manipulation, and because the rat prostatitis experimental model has been previously characterized. A strain of uropathogenic *Escherichia coli *(bacteria kindly provided by Dr. Pessah, Department of Microbiology, Cordoba University), isolated from patients with complicated urinary tract infection, was stored at -20°C and grown overnight in tryptic soy broth at 37°C when required for inoculations. Rats were anesthetized with inspired ether and subjected to laparotomy in order to expose the ventral prostate. Prostatitis was induced via injection of *E. coli *diluted in sterile PBS (200 μl total volume, 10^8 ^colony forming units per mL) or with the same volume of sterile PBS as a sham-infected control, using a 30 gauge needle inserted directly beneath the capsule of both prostate ventral lobes. The muscle, peritoneum and skin were then closed using a simple continuous pattern with chromic suture material. Three rats were sacrificed at 24, 48, or 72 hours after bacterial inoculation. Control, sham-infected rats were sacrificed 24 and 72 hours post-operation. No morphological differences were observed in the prostates obtained at the different time points in the control groups. The rat prostatitis model is based on a protocol published by Fulmer *et. al*. [29] with modifications as described by Quintar *et al*. [30]. This experiment was performed three times with 3 animals per group. Ventral prostates were either harvested and fixed in 4% paraformaldehyde and embedded in paraffin for light microscopy immunocytochemistry or frozen for biochemical analysis.

### Immunostaining of reproductive tissues

Paraffin-embedded mouse reproductive tissues or rat prostate tissues were sectioned (7 μm thick), deparaffinized and rehydrated serially in 100%, 95%, 75%, and 50% ethanol. For antigen retrieval, the slides were boiled in 0.1 M sodium citrate, pH 6.0, for 10 minutes and then cooled for 20 minutes. The slides were then rinsed in phosphate buffered saline (PBS) and endogenous peroxidase activity quenched by incubating in 0.3% H_2_O_2 _in PBS for 30 minutes. Slides were rinsed with PBS and blocked by incubating in normal serum (1.5%) for 20 minutes. The slides were then incubated with a SP-D polyclonal primary antibody (rabbit anti-mouse SP-D, which cross-reacts with rat SP-D, Chemicon, Temecula, CA, 1:1000) at 4°C overnight. For negative controls, slides were incubated overnight with either PBS or with normal rabbit IgG instead of the primary antibody. The slides were rinsed in PBS and incubated in secondary antibody (anti-rabbit IgG conjugated to biotin, Cappel, Aurora, OH) for 30 minutes at room temperature. Slides were rinsed, incubated in Vectastain ABC reagent (Vector Labs, Burlingame, CA) for one hour at room temperature, rinsed again in PBS and then incubated in diaminobenzidine (0.7 mg/mL), a peroxidase substrate. After a final PBS rinse followed by a H_2_O rinse, the slides were counterstained with hematoxylin, dehydrated and mounted using Permount. As a positive control for the SP-D staining, adult mouse lung tissue sections were also immunostained using the SP-D antibody.

### Immunoblot analysis

Rat prostate tissues (3 animals for each condition; i.e., sham, 24 hr, 48 hr and 72 hr) were homogenized in 1 mM phenylmethyl-sufonyl fluoride, centrifuged at 600 × g and the supernatant collected. Protein content was determined using a Bio-Rad Protein Assay (Bio-Rad Laboratories, Hercules, CA). Equal amounts of rat prostrate homogenate proteins (40 μg) were separated by gel electrophoresis on 15% Tris-HCl polyacrylamide gels. The proteins were transferred electrophoretically at 100 V to nitrocellulose membranes and then placed in 7% non-fat dry milk diluted in 0.1% TNT (0.02 M Tris, 0.15 M NaCl, 0.1% Tween 20) overnight to block non-specific binding. The membranes were subsequently incubated with the rabbit anti-SP-D primary antibody (Chemicon) at a dilution of 1:1000 for 1 hour at room temperature, then washed in 0.1% TNT, three times, at 15 minutes per wash. The blots were incubated with a secondary antibody (anti-rabbit IgG conjugated to horseradish peroxidase, 1:10,000 Cappel for 45 minutes at room temperature, then washed in 0.1% TNT, three times at 15 minutes per wash, and finally incubated with enhanced chemi-luminescence solution (Amersham, Buckinghamshire, UK) followed by exposure to X-ray film. Densitometry was then performed on the immunoreactive bands using Scion Image Software (Frederick, MD).

### Real time RT-PCR analysis

RNA was extracted from the male reproductive tract organs of 5–6 mice and also from the prostate and lung tissues of 9 castrated and 9 sham-castrated mice by homogenizing the tissues in Trizol (Invitrogen, Carlsbad, CA). RNA was extracted using chloroform and precipitated with ice-cold isopropanol. The resulting total RNA pellet was resuspended in water and quantitated by determining the absorbance at 260 nm. Two μg of total RNA from each sample were then reversed transcribed. The resulting cDNAs were diluted (1/50) and replicates for each sample were aliquoted for real time polymerase chain reaction (PCR) analysis using a Stratogene Mx 3000 P instrument. FAM-labeled primers for mouse SP-D and 18 S ribosomal RNA (the transcriptional product of a housekeeping gene) and Universal Taqman master mix were purchased from Applied Biosystems, Inc. (ABI, Foster City, CA). As a control, the threshold cycle (CT) values obtained from adding increasing amounts of RNA with either SP-D primers or 18 S primers were plotted. The two primer sets produced parallel curves with similar slopes. SP-D mRNA levels were normalized to 18 S rRNA levels and then the relative SP-D mRNA levels were determined by the comparative quantitation method, according to the manufacturer User Bulletin 2 (10/2001, ABI systems). For experiments in which the levels of SP-D mRNA in different mouse male reproductive organs were compared, the levels were expressed relative to the average level of SP-D mRNA in the prostate, which was made equal to one. For experiments in which levels of SP-D were compared in prostate and lung tissue from intact vs. castrated mice, SP-D mRNA levels are expressed relative to levels in intact tissues from sham-castrated control mice.

### Statistics

All data were derived from at least three experiments. The data were analyzed by one-way analysis of variance followed by Student-Newman-Keuls Multiple Comparisons Test.

## Results

### SP-D is present in the mouse male reproductive tract

**Figure 1:**
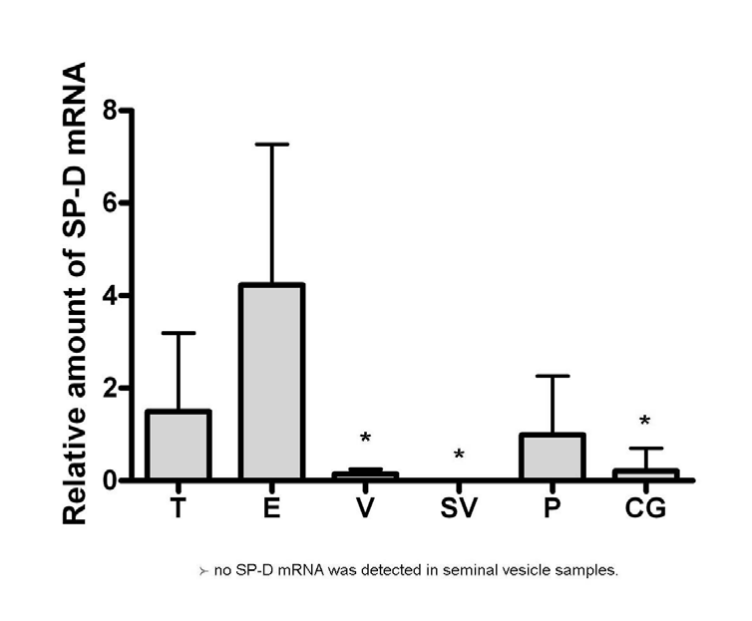
**Relative amount of SP-D mRNA in mouse male reproductive tract tissues as determined by semi-quantitative real time RT-PCR**. T = testis, E = epididymis, V = vas deferens, SV = seminal vesicle, P = prostate, and CG = coagulating gland. SP-D mRNA was detected in the testis, epididymis, vas defrens, prostate, and coagulating gland but was not detectable in the seminal vesicle. The data represent the mean, plus or minus the standard deviation, n = 5–6 animals. An asterisk denotes a significant decrease in SP-D mRNA levels in the vas deferens, seminal vesicle, and coagulating gland as compared to the epididymis, ANOVA, p < 0.05.

To determine if SP-D is produced throughout the mouse male reproductive tract, real time RT-PCR was performed on RNA isolated from several male reproductive tract tissues, i.e., testis, epididymis, vas deferens, seminal vesicle, prostate, and coagulating gland. SP-D mRNA was detected in all of the tissues except for the seminal vesicle (Figure 1). The levels of SP-D mRNA in the male reproductive tract were considerably lower than the levels detected in the lung, ~2,000 fold less (data not shown). The levels of SP-D mRNA did not differ significantly between the testes, epididymis and prostate. SP-D mRNA in the coagulating gland and vas deferens were detectable but the levels were lower than in the epididymis.

**Figure 2:**
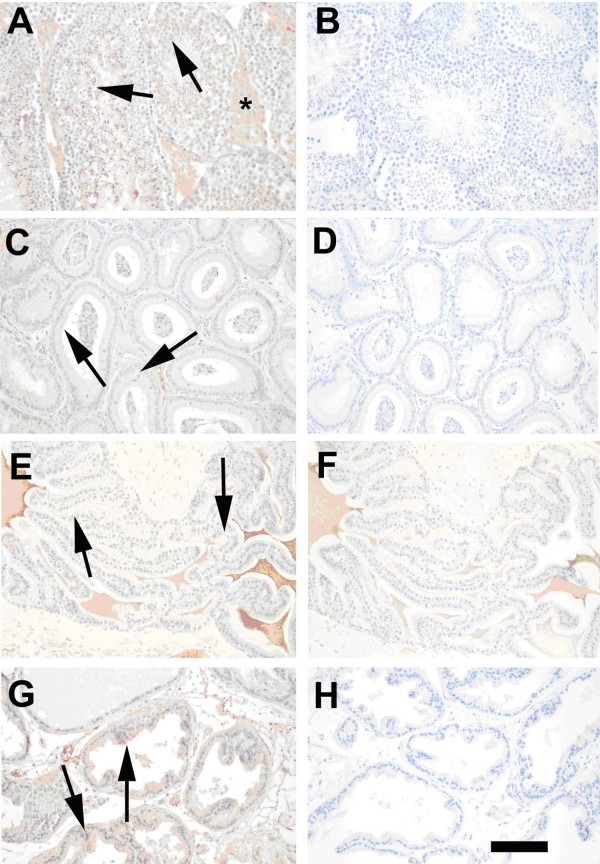
**SP-D immunostaining in mouse male reproductive tissues**. Photographs in the left column are tissues immunostained for SP-D. Corresponding photographs on the right are the same tissues immunostained using normal rabbit IgG instead of the primary antibody. A and B. Testis. The interstitial cells (asterisks) of the testis stained positively for SP-D protein. Sertoli cells and spermatids also stained positively (arrows). C and D. Epididymis. SP-D immunostaining was observed in the epithelium of the epididymis (arrows). E and F. Seminal vesicle. Almost no SP-D immunostaining was observed in the seminal vesicle. Secreted material in the lumen of the gland was stained non-specifically in both the immunostained sections and in the normal IgG control sections. G and H. Prostate. SP-D immunostaining was present in the epithelium of the prostate (arrows). Reproductive tissues obtained from at least three different mice were immunostained with similar results. Magnification bar = 100 μm.

In the testes, peritubular tissues including vascular endothelium and some interstitial cells (asterisks), stained strongly for SP-D protein, however Sertoli cells and spermatids also stained positively (Figure 2A, arrows). The epithelial cells of the epididymis stained weakly positively for SP-D protein (Figure 2C, arrows). In agreement with the real time RT-PCR data, immunoreactive SP-D was present in very low levels in the seminal vesicle (Figure 2E). SP-D protein was detected in the epithelium of the mouse prostate (Figure 2G, arrows).

### Castration alters SP-D expression in the mouse prostate

**Figure 3:**
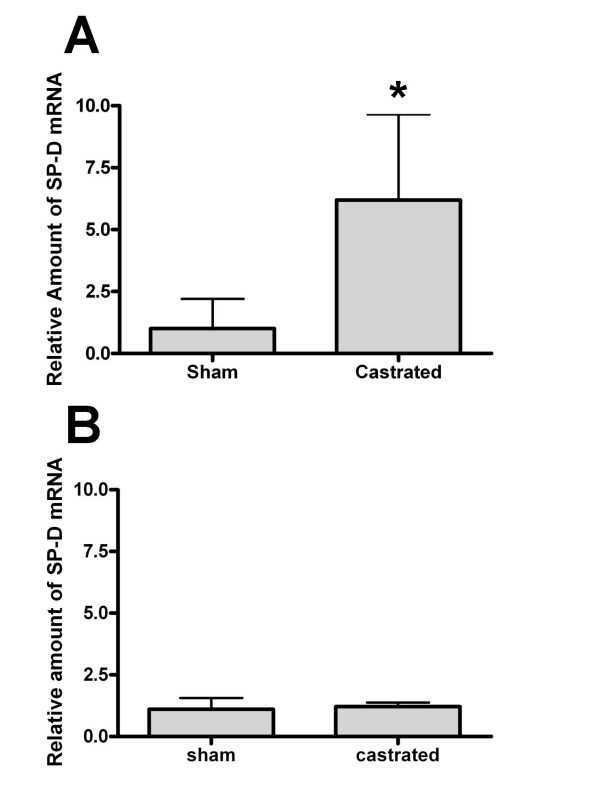
**Relative amount of SP-D mRNA in prostate (panel A) or lung tissue (panel B) from sham-castrated or castrated animals as determined by semi-quantitative real time RT-PCR**. A. Prostate tissue. There was a significant increase in SP-D mRNA in the prostates of mice that were castrated when compared to sham-castrated mice. B. Lung tissue. SP-D lung mRNA levels were not affected by castration. The data represent the mean plus or minus the standard deviation, n = 9 sham-castrated animals and n = 9 castrated animals. The asterisk denotes a significant difference between the castrated and the control, sham-castrated animals, ANOVA, p < 0.05.

Testosterone affects the growth of the prostate gland and other organs in the male genital tract [5]. To determine if androgens regulate SP-D expression in the prostate, mice were castrated or sham-castrated. The mice were then sacrificed 12 days post-surgery, their prostates and lungs removed, and SP-D mRNA levels analyzed (Figure 3). The levels of SP-D mRNA in prostates of castrated mice were increased significantly (~7 fold) when compared to levels in mice that were sham-castrated (Figure 3A). SP-D mRNA levels in the lungs from these mice; i.e., sham-castrated and castrated, were not different from each other (Figure 3B). We also examined SP-D mRNA levels in other parts of the male urogenital tract; i.e., the epididymis, and the bladder and observed no change in SP-D mRNA levels with castration (data not shown).

### Rat prostatitis causes an increase in SP-D expression

**Figure 4:**
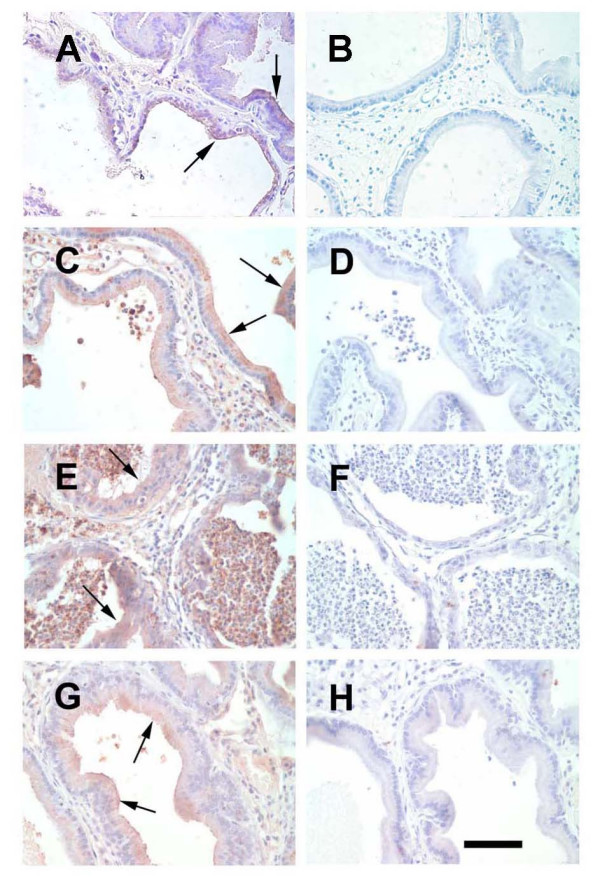
**SP-D immunostaining of rat prostate tissue**. Rats were either sham-infected or infected with *E. coli*. Prostates were removed 24, 48, and 72 hours post-infection. Photographs in the left column are SP-D immunostaining and the corresponding photograph on the right is the same tissue immunostained using normal rabbit IgG as a staining control. A and B. Sham-infected prostate. The rat prostate epithelium contains SP-D protein with the staining present primarily on the apical portion of the epithelial cells (arrows). C and D. Rat prostate 24 hours post-infection. The epithelial SP-D staining is present throughout the cytoplasm of the epithelial cells (arrows). E and F. Rat prostate 48 hours post-infection. Many neutrophils were present in the lumen of the prostate glands at this time. The cytoplasm of the prostate epithelium stained positive for SP-D protein (arrows) and SP-D is present in the lumen and within neutrophils as well. G and H. Rat prostate 72 hours post-infection. SP-D staining intensity remained high in some epithelial cells (arrows). Magnification bar = 100 μm.

**Figure 5:**
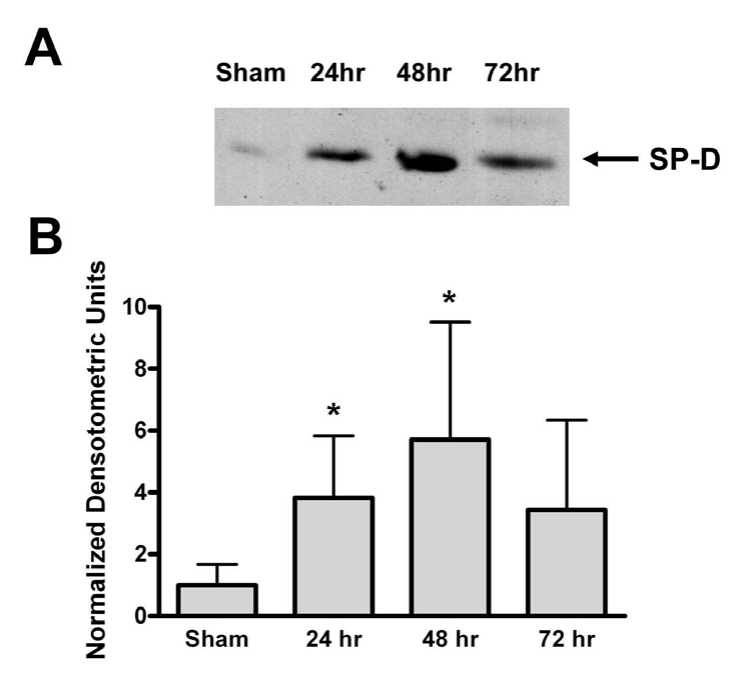
**SP-D immunoblot of rat prostate tissue**. A. A representative SP-D immunoblot of infected rat prostate tissues. The first lane contains rat prostate protein homogenate (40 μg) from a sham-infected animal, the last three lanes contain protein homogenate (40 μg) from rat prostates that were infected with *E. coli *and harvested 24, 48 and 72 hours post-infection, respectively. Immunoreactive SP-D migrated at a molecular weight of ~40 kDa (arrow). B. Densitometric analysis of SP-D immunoblots. SP-D protein levels were significantly increased at 24 and 48 hours post-infection when compared to the levels in sham-infected controls, which were equal to one. SP-D levels from tissues obtained 72 hours post-infection were not significantly different from control levels. This experiment was repeated three times, n = 3 animals per condition. The asterisk denotes a significant difference between the experimental condition and the control, sham-infected animals, ANOVA, p < 0.05.

SP-D protects against infection in the lung and pulmonary SP-D levels are known to be altered by infection and other disease states[24]. Previous work from our laboratory indicated that SP-D can inhibit chlamydial infection of prostate epithelial cells *in vitro *[23]. Therefore, we wished to determine if SP-D protein expression is altered with prostate infection. A rat model of prostatitis achieved via direct infection with *E. coli *was used to address this question. Infected rats were sacrificed 24, 48 and 72 hours post-infection and their prostates removed. As a control, some rats were injected with the vehicle alone (sham-infected). SP-D protein was present in the epithelium of the prostate in sham-infected rats, primarily in the apical portion of the cells (arrows, Figure 4A). Epithelial cell SP-D staining intensity was increased in the prostate of *E. coli *infected rats at 24 and 48 hours post-infection (arrows, Figure 4C and 4E). Many SP-D positive neutrophils were present in the lumen of the prostatic ducts of infected animals at 24 and 48 hours. By 72 hours post-infection, the overall level of SP-D staining in the prostate epithelium of infected rats was less intense; however, the staining intensity remained elevated when compared to levels in the sham-infected animals (Figure 4G). We also evaluated SP-D protein levels in the prostate tissue at the various time points after infection using immunoblot analysis (Figure 5A). Immunoreactive SP-D in the rat prostate migrated at ~40 kDa, the previously reported molecular weight of rat SP-D [31]. SP-D protein levels were significantly increased in prostate tissue obtained from rats 24 and 48 hours post-infection. However, by 72 hours post-infection the levels of SP-D protein had declined and were not different from controls (Figure 5B).

## Discussion

Innate immune proteins have primarily been characterized in myeloid cells and in epithelial cells continuously exposed to pathogens such as in the lung and digestive tract. More recently, several investigators have demonstrated the importance of the innate immune system in the reproductive tract [8,32-36]. In the male urogenital system, investigations about innate immunity have focused particularly on the epididymis [35,36] and testis [37]. In contrast, local defense mechanisms protecting the prostate gland are poorly understood.

SP-D has been proposed as a central player in innate host defense [9,24]. Therefore, we were interested in investigating the presence of SP-D in the murine reproductive tract. In this study, we show that SP-D mRNA and protein are expressed in several organs in the mouse male genital tract, including in the prostate. SP-D is localized to the epithelium of the prostate, epididymis, vas deferens, testes, and is also detected in interstitial cells of the testis. The seminal vesicle was the only male reproductive tract organ that did not contain significant amounts of SP-D. The relative level of SP-D mRNA detected in the male reproductive tract is much lower, ~2,000 fold less, than observed in the lung. However, since the proportion of epithelium in the male reproductive tract organs is substantially less than in the lung, local concentrations of SP-D on the mucosal surfaces of the male reproductive tract may not be as low as the real time RT-PCR data indicate. Because of the lack of SP-D expression in seminal vesicles, which are the major contributors to seminal plasma, it is possible that prostate-secreted SP-D may be an important antimicrobial factor that accompanies spermatozoa in semen.

Several authors have previously shown that SP-D protein levels can be increased by infection. For instance, SP-D protein was increased in human gastric mucosa during infection with *Helicobacter pylori *[38]. *Pneumocystis carinii *infection has also been shown to increase SP-D levels in the mouse lung [39]. Serum levels of SP-D were increased in mice with acute and chromic inflammation of the lung [40]. We choose to study the influence of infection using a rat model of prostatitis because the rat prostate is a larger size, which allows for easier manipulation when compared to the mouse prostate (Quintar *et al*., 2006). The ventral prostate of the mouse and rat are anatomically and functionally similar [41,42]. The gross morphology of the prostate glands is essentially identical in the two species, although the rat prostate gland is larger and more complex [41]. The morphogenesis of the prostate gland in the two species is also very similar [42]. Both the mouse and rat prostates are used as animal models for studying prostatitis and prostate cancer [43-45]. In the present study, we demonstrate that *E. coli *infection stimulates SP-D protein production in the rat prostate epithelium. Prostate SP-D protein levels were significantly increased early in the prostate infection, i.e., 24 hours and 48 hours post-infection. However, by 72 hours post-infection, SP-D levels in the infected rat prostate epithelium had declined. This pattern of expression was expected since components of the innate immune system are often the first responders to infection.

Cytokines have previously been shown to regulate SP-D expression [28]. We have recently reported that increased levels of SP-D were correlated with inflammation in the human prostate gland [23]. In the rat prostatitis model used in the current study, we observed a striking increase in neutrophils within the gland by 48 hours post-infection, which corresponded to the peak of SP-D expression. Since SP-D has anti-inflammatory properties, we hypothesize that SP-D may increase at sites of inflammation in part to dampen the immune response [46]. However, SP-D levels could also have been stimulated directly by the presence of a pathogen and this in turn may cause the recruitment of inflammatory cells to the site of infection. LPS instillation in the lung has shown to produce an increase in the expression of SP-D [25]. Thus, increased SP-D expression in the rat prostate might have been induced by *E. coli *LPS. Since SP-D agglutinates bacteria, promotes phagocytosis and in some cases has direct anti-microbial affects on certain strains of *E. coli*, it may be beneficial for the host to increase expression of SP-D soon after an infection has begun [17,24]. Consequently, SP-D modulation within the prostate gland may be a key mechanism to guarantee effective clearing of microorganisms, in this way preventing the progression of infection toward more restricted sites of the male reproductive tract such as the epididymis and the testis. Interestingly, we detected immunoreactive SP-D protein in neutrophils present in the ducts of the infected prostate. Other investigators have shown that SP-D is taken up by neutrophils during inflammation in the lung [47].

Androgen withdrawal leads to involution of the prostate gland with alterations that include a decrease in secretion and loss of epithelial cells by apoptosis. In the present study, we found that castrated mice have increased SP-D mRNA in their prostates. Interestingly, castration of the male mouse did not change SP-D mRNA levels in the lung or in other tissues in the male reproductive tract of the mouse. Although hormonal regulation of SP-D by growth hormone has been previously reported, this is the first study to suggest that SP-D can be regulated by androgens [48]. In a recent paper by Sorensen *et al*., it was shown that SP-D levels in serum increase with age in humans and that SP-D levels are higher in males than in females [49]. These data are consistent with the concept that hormones may play a role in regulating SP-D levels. It is well established that glucocorticoids stimulate SP-D mRNA expression in the lung; however, the mechanism involved is not fully understood [50]. It is also known that glucocorticoids increase the expression of glucocorticoid receptor, whereas androgens decrease glucocorticoid receptor levels in the lung, ovaries, prostate and liver [51]. Therefore, it is possible that androgens may regulate prostate SP-D expression indirectly through the glucocorticoid receptor pathway.

SP-D appears to be particularly important in apoptotic cell removal from the lung [52]. Thus, it is conceivable that the increased SP-D levels in the prostate after castration may be involved in removal of the increased apoptotic cells that follow testosterone deprivation.

## Conclusion

In summary, we detected SP-D mRNA and protein throughout the mouse male genital tract, including in the prostate. We determined that SP-D expression in the prostate is increased in castrated male mice. We also demonstrated that prostate SP-D gene expression increases in response to infection. These data are the first to suggest a role for SP-D in prostatic innate immunity *in vivo *and the first to show that prostatic SP-D may be regulated by androgens, findings that may lead to a better understanding of innate immunity in the prostate gland.

## Competing interests

The author(s) declare that they have no competing interests.

## Authors' contributions

All authors of this manuscript contributed to the paper by conducting the research directly, writing portions of the manuscript, or contributing to the ideas behind the different projects involved in this manuscript. REO is the primary author of this paper and helped conduct SP-D immunoblotting and immunostaining and castration experiments. KLG helped conduct SP-D immunoblotting, immunostaining and RT-PCR. AAQ and CAM conducted the prostatitis experiments and helped write some of the manuscript. JMS is the PI of the laboratory and helped conduct the SP-D RT-PCR experiments and also helped write parts of the manuscript. All authors have read and approved the final manuscript.
